# Pancreatic morphological abnormality that was challenging to differentiate from an ectopic pancreas: a case report

**DOI:** 10.1186/s40792-022-01404-x

**Published:** 2022-03-28

**Authors:** Takuto Yoshida, Hideki Kawamura, Kazuhiro Mino, Yuji Konishi, Tomoya Saito, Yuichi Shimizu, Akinobu Taketomi

**Affiliations:** 1 Department of General Surgery, Hokkaido Medical Center, 1-1, 5-7, Yamanote, Nishi-ku, Sapporo, 063-0005 Japan; 2 Department of Gastroenterology, Hokkaido Medical Center, 1-1, 5-7 Yamanote, Nishi-ku, Sapporo, 063-0005 Japan; 3 Department of Gastroenterological Surgery 1, Graduate School of Medicine, Hokkaido University, N-15, W-7, Kita-ku, Sapporo, 060-8638 Japan

**Keywords:** Protrusion of the lateral contour of the pancreatic head, Normal variations in pancreatic contour, Ectopic pancreas

## Abstract

**Background:**

Protrusion of the lateral contour of the pancreatic head is a pancreatic morphological abnormality, which is known as rare shape atypia. We present a rare case of protrusion of the lateral contour of the pancreatic head, which was challenging to distinguish from an ectopic pancreas.

**Case presentation:**

The patient was a 40-year-old man with a history of acute pancreatitis that occurred twice in the past. He complained of epigastric pain since the day before the visit; his blood workup showed high serum amylase level and a CT scan revealed a 25-mm-large mass with contrast effect from the anterior wall of the gastric pylorus to the duodenum and increased surrounding fatty tissue density. Endoscopic ultrasonography revealed a mass lesion in the gastric pylorus with continuity with the gastric wall and suspected partial continuity with the pancreatic head. Thus, the possibility of pancreatic morphological abnormality or an ectopic pancreas was considered. Following which, resection was attempted and intraoperative findings showed a wide extension of the pancreatic parenchyma from the pancreatic head to the anterior wall of the gastric pylorus to the duodenal bulb. Since the patient only had mild pancreatitis, the resection was judged to be too invasive and was completed by exploratory laparoscopy.

**Conclusions:**

Even if the findings on preoperative CT are suspicious for an ectopic pancreas or tumor, a pancreatic morphological abnormality, such as a protrusion of the lateral contour of the pancreatic head, should be included in the differential diagnosis.

## Background

Protrusion of the lateral contour of the pancreatic head is a rare pancreatic morphological abnormality and is sometimes difficult to distinguish from a pancreatic tumor or ectopic pancreas. We present a case of protrusion of the lateral contour of the pancreatic head, which was discovered during a close examination for repeated pancreatitis and was challenging to differentiate from an ectopic pancreas.

## Case presentation

A 40-year-old man with a history of acute pancreatitis, which occurred twice in the past without any apparent cause, presented with epigastric pain experienced since the day before the visit. The patient’s blood workup showed a high level of serum amylase. He was suspected of having acute pancreatitis and was therefore referred to our hospital. The patient’s history and his family history was unremarkable, other than a history of acute pancreatitis, and he had no history of heavy alcohol consumption or regular medication. Physical examination revealed tenderness in his upper abdomen, and blood tests showed white blood cell count of 6600 × 103/μl, C-reactive protein of 0.27 mg/dl, triglyceride of 148 mg/dl, amylase of 135 U/l, P-amylase of 83 U/l, and lipase of 67 U/l.

**Fig. 1:**
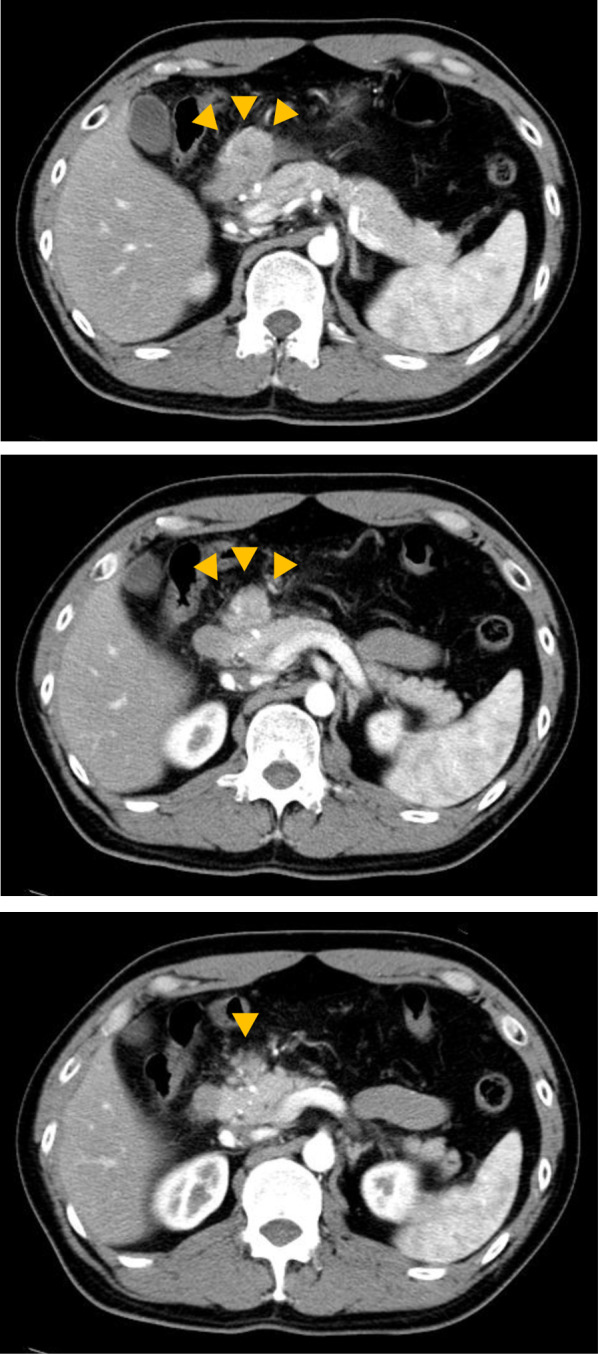
Findings of abdominal computed tomography. CT revealed a 25-mm-large mass lesion with contrast effect from the anterior wall of the gastric pylorus to the duodenum (yellow arrow). From the CT image, it is difficult to distinguish the continuity between the tumor and the pancreatic head, which looks more like a tumor. *CT* computed tomography

**Fig. 2:**
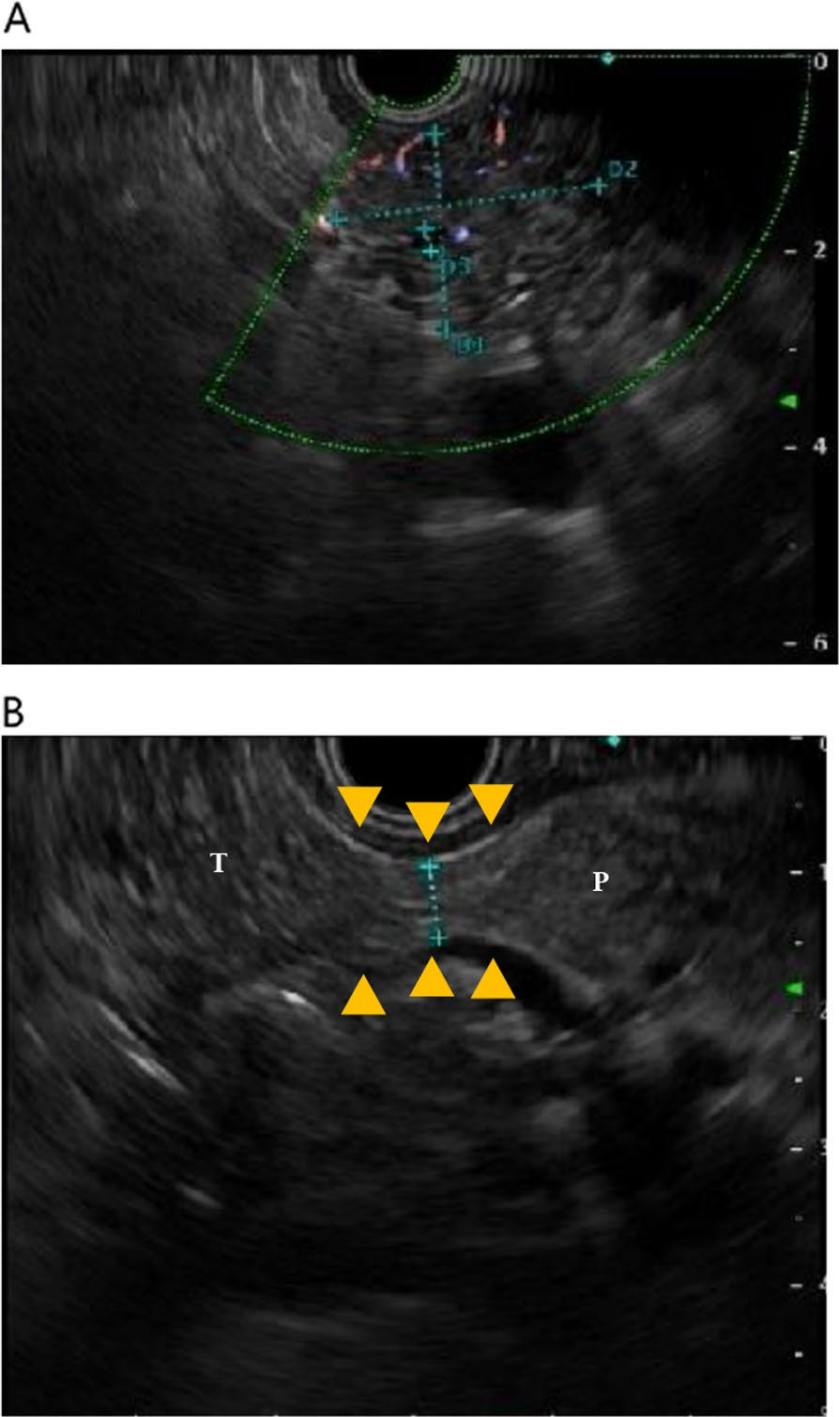
Findings of endoscopic ultrasonography (EUS). **A** EUS revealed a mass-like lesion in the gastric pylorus with continuity with the gastric wall, which was 26.7 × 20.0 mm in size. **B** The tumorous lesion (T) was suspected to be continuous with the pancreatic parenchyma (P) with a partial thickness of approximately 5 mm (yellow arrow)

**Fig. 3:**
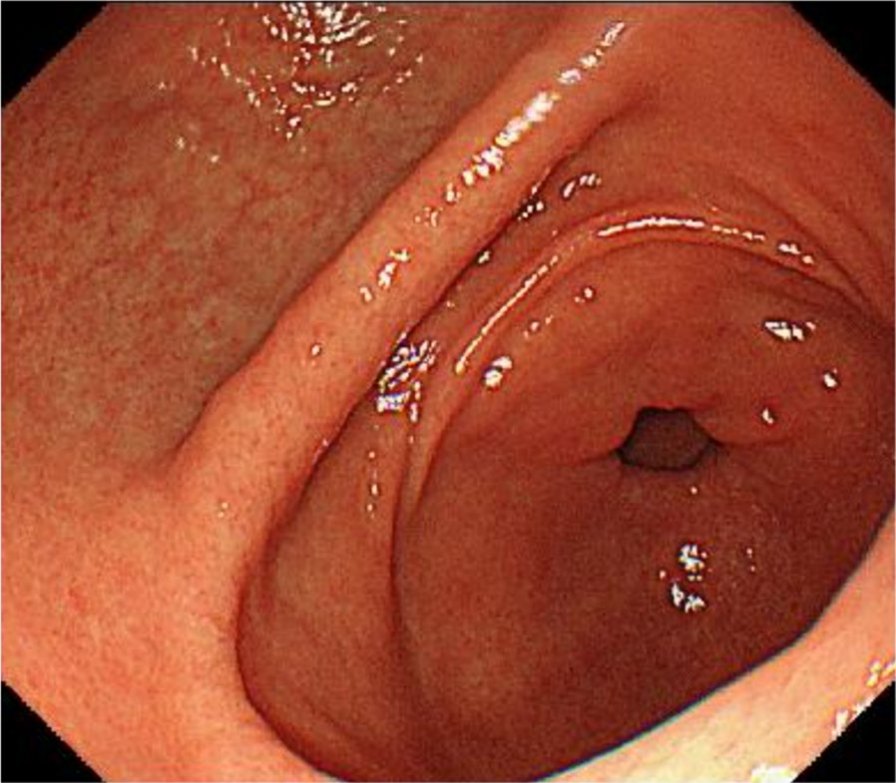
Findings of upper gastrointestinal endoscopy. The endoscopic findings were normal and did not show an submucosal tumor like mass as seen in an ectopic pancreas

**Fig. 4:**
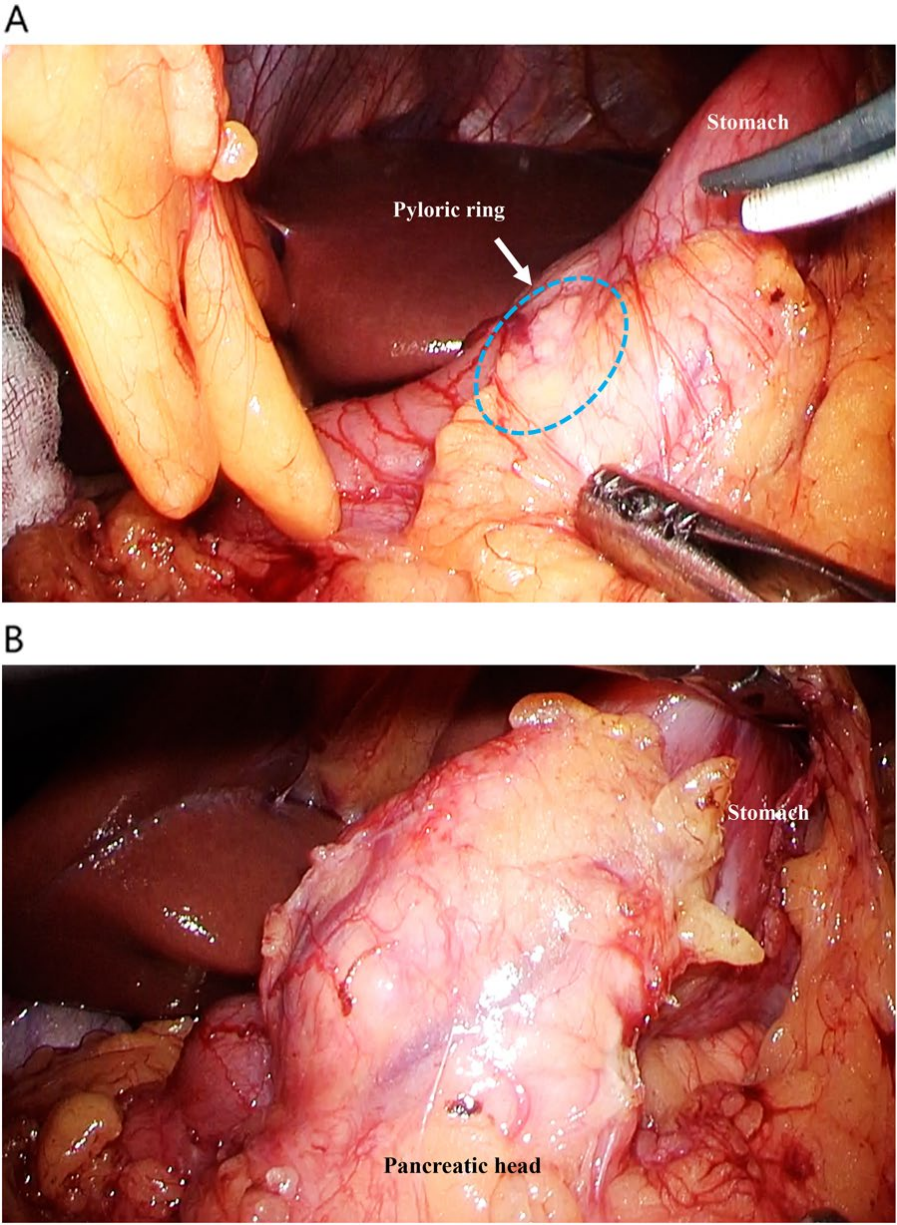
Intraoperative findings. **A** Intraoperative findings showed a broad extension of the pancreatic parenchyma from the pancreatic head to the anterior gastric side of the gastric pylorus to the duodenal bulb. Slight inflammatory changes were observed around the pyloric ring (blue dotted circle). **B** The great omentum was resected to confirm the continuity between the pancreatic head and the pancreatic tissue extending to the pyloric ring

Contrast-enhanced computed tomography (CT) revealed a 25-mm-large mass-like lesion with contrast effect from the anterior wall of the gastric pylorus to the duodenum (Fig. 1) and elevated surrounding fatty tissue density, leading to the diagnosis of acute pancreatitis due to ectopic pancreas. Although pancreatitis disappeared in a few days by implementing fasting and administering antibiotics, the patient continued to have similar episodes; thus, he visited our department. Preoperative endoscopic ultrasound (EUS) revealed a mass-like lesion in the gastric pylorus with continuity in the gastric wall, which was 26.7 × 20.0 mm in size (Fig. 2A). We did not perform fine needle aspiration at this time. Although EUS showed a suspected continuity between the tumor and the pancreatic head with a partial thickness of ~ 5 mm (Fig. 2B), the CT scan showed no continuity. Since the tumor was contrasted in a pattern similar to that of the pancreas and the ectopic pancreas was predominantly located in the pyloric region, ectopic pancreas and pancreatic morphological abnormality were considered differential diagnoses. In addition, there was no noticeable thickening of the bile duct wall, stones, or pancreaticobiliary maljunction. No obvious abnormality was found in upper gastrointestinal endoscopy or barium contrast X-ray examination (Fig. 3). Based on the above, the patient was scheduled for a laparoscopic distal gastrectomy to resect the ectopic pancreas. Intraoperative findings revealed a non-pedunculated broad extension of the pancreatic parenchyma from the pancreatic head to the anterior wall of the gastric pylorus to the duodenal bulb (Fig. 4A, B). We did not perform intraoperative ultrasonography and intraoperative biopsy because we did not actively suspect a tumor and were concerned about the risk of pancreatic fistula. We dissected the greater omentum during the operation to expose the pancreatic tissue completely, thereby confirming its continuity with the pancreas. There was no apparent ectopic pancreas or mass lesion in any other location. Since the resection required extensive cutting of the pancreatic parenchyma and the patient had only mild pancreatitis, the resection was judged to be excessively invasive and was completed only by exploratory laparoscopy. The postoperative period was uneventful, and the patient was discharged on postoperative day 4. Fortunately, his pancreatitis has not flared up so far.

## Discussion

Morphological abnormalities of the pancreas include pancreatic divisum, annular pancreatic agenesis/hypogenesis of the body and tail of the pancreas, ectopic pancreas, protrusion of the lateral contour of the pancreatic head and pancreatic tail, intrapancreatic parasplenium, and splenic varices [1–3]. It is important to differentiate these from pancreatic tumors. Protrusion of the lateral contour of the pancreatic head includes a convex protrusion of the lateral margin of the pancreatic head and a beak-shaped protrusion of the uncinate process. Ross et al. reported that dual-phase helical CT was performed in 119 patients without pancreatic disease; of which, 41 patients (34.5%) had a tissue that protruded more than 1 cm laterally from the gastroduodenal artery or the anterior superior pancreaticoduodenal artery [4]. Contour variants of the pancreatic head and neck are classified into three types according to the direction of protrusion based on the gastroduodenal artery or the anterior superior pancreaticoduodenal artery; the three types are as follows: anterior type (type 1), posterior type (type 2), and horizontal type (type 3). On the other hand, Chandra et al. found that 12 of 21 patients (57%) with intestinal malrotation had an abnormal pancreatic head morphology, which could be classified into three categories: globular-shaped (24%), elongated (24%), and combined globular and elongated type (10%) [5]. Since the pancreatic development is closely related to the development of the intestinal tract, it has been reported that pancreatic morphological abnormality may be more common in patients with abnormal intestinal rotation. The cause of the protrusion of the lateral contour of the pancreatic head is considered to be related to the pattern of fusion of the ventral and dorsal pancreatic primordium. The dorsal and ventral pancreatic primordia arise from the duodenum at 4 weeks of fetal life [6]. At 5 weeks of fetal life, when the duodenal primordium rotates in a clockwise direction, the ventral pancreatic primordium rotates clockwise with the primitive common bile duct, and migrates below the dorsal pancreatic primordium. At 6 weeks of fetal life, the dorsal and ventral pancreatic primordium begin to fuse. At 7 weeks of fetal life, the fusion is completed to form the ventral pancreatic duct, and the proximal part of the dorsal pancreatic duct becomes the peripancreatic duct. If there are doubts regarding the diagnosis, it is essential to confirm the contrast pattern of the normal pancreatic parenchyma by multiphase, thin-slice, contrast-enhanced CT to accurately differentiate morphological abnormalities from neoplastic lesions [7]. Aysel et al. reported that magnetic resonance cholangiopancreatography (MRCP) can capture abnormalities in the pancreatic duct travel and is useful in diagnosing pancreatic morphological abnormalities [1]. If a non-pedunculated broad continuity could be confirmed by imaging studies including EUS, it would have been possible to diagnose pancreatic morphological abnormalities preoperatively. It is possible that only the uniaxial direction was visible on the EUS images. Although the most common type of protrusion of the lateral border of the pancreas is the posterior type (type 2), this case is considered the anterior type (type 1). The ectopic pancreas, which is listed as a differential diagnosis, refers to the pancreatic tissue existing in other organs without continuity with the original pancreas. The ectopic pancreas is found in 0.11–21% of autopsy cases and is preferentially located in the stomach, duodenum, and air conditioning close to the pancreatic base [8]. In the stomach, 88% of cases are reported to be located in the pyloric vestibule and 12% in the body [9]. When they occur in the lumen of the gastrointestinal tract, they may enlarge and cause obstruction or bleeding, resulting in abdominal discomfort and epigastric pain. Although ultrasound endoscopy and ultrasound endoscopic aspiration cytology are used for diagnosing ectopic pancreas, the positive diagnostic rate of ectopic pancreas by EUS-fine needle aspiration is not necessarily high (50–80%) [10–12]. Adenocarcinoma in ectopic pancreas is rare (0.7–1.8%) [13, 14] and most of the cases are asymptomatic and can be followed up. Still, surgery is considered for symptomatic patients. A definitive diagnosis cannot be made, and it is necessary to differentiate between gastrointestinal stromal tumor, cancer, insulinoma, and cases in which there is a tendency for the ectopic pancreas to increase in size [15]. In this case, the indication for surgery was appropriate owing to the mild but recurring pancreatitis. However, since there was a discrepancy in the thickness of the continuity between the tumor and the pancreatic head in the preoperative EUS and intraoperative findings, it might have been better to consider biopsy. Moreover, we did not rule out malignancy by biopsy before the surgery; thus, it may have been necessary to perform positron emission tomography–CT.

A search on PubMed using the keywords "pancreas" and "protrusion of lateral contour" revealed no case reports, suggesting that protrusion of the lateral border of the pancreatic head in the anterior type (type 1) is an extremely rare condition, and there is no description of its relationship with pancreatitis. Therefore, the apparent cause of pancreatitis in this case is unknown. Intraoperative findings revealed grossly visible changes that suggested only mild inflammation at the margins of the pancreatic tissue extending into the duodenum. If the pancreatic morphology abnormality is a matter of fusion between the dorsal and ventral pancreatic primordium, the pathology may resemble pancreatitis associated with pancreas divisum [16]. In other words, in pancreas divisum, in which the dorsal and ventral pancreatic ducts perform their excretory functions independently, pancreatitis may develop due to the relationship between the size of the opening and the amount of pancreatic juice to be drained. In general, the opening of the dorsal pancreatic duct into the accessory papillae as a main pancreatic duct is often a problem, and it is considered that such an abnormal running of the pancreatic duct may have been related to the pathogenesis in this case as well. MRCP or Endoscopic retrograde cholangiopancreatography would have been necessary to obtain this definitive diagnosis. As for the choice of surgical procedure, partial resection is considered the first choice when operating on an ectopic pancreas. In this case, we preoperatively judged that partial resection would be difficult because of a broad extension of the tumor from the anterior gastric side of the gastric pylorus to the duodenal bulb and planned to perform a distal gastrectomy including the duodenum where the mass was located. In addition, the possibility of a pancreatic morphological abnormality was also considered because preoperative EUS suspected the continuity of a 5 mm-diameter mass with the pancreatic head. In case of abnormal pancreatic morphology, if the diameter was small and could be resected safely, distal gastrectomy with resection of the pancreatic parenchyma could be performed. Based on the intraoperative findings, it was considered that the present case was not an ectopic pancreas but a thick-diameter protrusion of the lateral contour of the pancreas and that the risk of resection and invasiveness was high compared to the past episodes of mild pancreatitis. We therefore decided to terminate the surgery with observation only. In cases of pancreatic tumors or ectopic pancreas, which are difficult to diagnose in preoperative examinations, it is necessary to determine a treatment plan keeping in mind pancreatic morphological abnormalities such as protrusion of the lateral contour of the pancreatic head.

## Conclusion

We were presented with a case of abnormal pancreatic morphology that is very similar to an ectopic pancreas. When imaging tests reveal neoplastic lesions, such as ectopic pancreas, pancreatic morphological abnormalities, such as protrusion of the lateral border of the pancreatic head, should be considered. In this case, we only observed the case without performing a resection surgery, because the inflammatory findings of the pancreas were minor, and there was continuity between the pancreatic head and the thickened broad base.

## Data Availability

The dataset supporting the conclusions of this article is included in the article.

## Abbreviations

CT: Computed tomography
EUS: Endoscopic ultrasound
MRCP: Magnetic resonance cholangiopancreatography
